# Landscape, Environmental, and Socioeconomic Impacts of an Invasive Bird Species: The Yellow-Legged Gull (*Larus michahellis*) in the Natural Park Salinas de San Pedro del Pinatar (Murcia, Southeastern Spain)

**DOI:** 10.3390/life15030361

**Published:** 2025-02-25

**Authors:** Gustavo Ballesteros-Pelegrín, Miguel Ángel Sánchez-Sánchez, Alfonso Albacete

**Affiliations:** 1 Department of Geography, Autonomous University of Madrid, 28049 Madrid, Spain; gustavo.ballesteros@uam.es; 2 Department of Geography, University of Murcia, 30001 Murcia, Spain; miguelangel.sanchez2@um.es; 3 Institute of Agroenvironmental Research and Development of Murcia (IMIDA), 30150 Murcia, Spain

**Keywords:** yellow-legged gull, management, habitat, waterfowl, salt production, salt workers

## Abstract

The yellow-legged gull (*Larus michahellis*) increased its population throughout the 20th century in its worldwide distribution area. In the Salinas de San Pedro del Pinatar, the population increased from having two breeding pairs in 1993 to 676 pairs in 2010 and from a wintering population of approximately 100–200 individuals in the 1980s to 1500–2000 individuals recorded in the 2010s, which has led to changes in habitats due to guano deposition, bird predation, incidents involving workers, and salt production. The objective of this study is to analyze the impacts of *L. michahellis* on the landscape, habitats, waterfowl, salt production, and workers, as well as to evaluate the effectiveness of control activities. Censuses of wintering *L. michahellis* have been carried out between 1990 and 2021, of nesting aquatic birds between 1994 and 2021, and nests and eggs of *L. michahellis* have been eliminated between 2000 and 2021. The result has been a decrease in pairs of *L. michahellis*, recovery of waterfowl populations, colonization of new bird species, absence of incidents with workers, and reduction in damage to salt production. Importantly, to reach a definitive solution, measures should be adopted to prevent *L. michahellis* from accessing the main sources of human food: urban solid waste dumps, aquaculture farms, and fish discards.

## 1. Introduction

The yellow-legged gull (*Larus michahellis* Naumann, 1840) (YLG) inhabits the Mediterranean coast of southern Europe, the Black Sea, and the Caspian Sea, as well as the Azores, Madeira, and the Canary Islands, the Iberian Peninsula, the Middle East, and North Africa [1]. The global population is unknown, but it shows an increasing trend both worldwide and in Europe [2]. The European population was estimated at between 409,000 and 534,000 pairs in 2015 [3], while the wintering population in Spain was estimated at around 230,000 individuals in 2010 [4] and recorded a minimum of 125,000 breeding pairs between 2007 and 2009 [5].

It is listed on the global and European Red Lists as of least concern [2], due to population growth driven by the use of open landfills, fishing discards, and marine aquaculture farms [4,6,7,8,9]. However, the 2022 Spanish Red Book classified it as near threatened due to a significant decline in population size, albeit heterogeneously, with a more marked reduction in the Cantabrian–Galician population of the subspecies *lusitanius* [10], mainly due to the closure of numerous landfills [9,11,12]. The species is included in Annex I of Royal Decree 1095/89, which declares it a game species, while the Bern Convention lists it in Annex III, where species subject to regulated exploitation are included [4].

In the Regional Park of the Salinas and Arenales of San Pedro del Pinatar, its population grew continuously from the first appearance of two pairs in 1993 to a peak of over 676 pairs in 2016 [10]. It is estimated that the population would likely exceed 1000 pairs if annual nest and egg removal efforts were not carried out.

Several studies have examined how the growing population and range of YLGs affect the Iberian Peninsula’s flora and fauna. These impacts include predation on chicks of both terrestrial birds (like partridges) and aquatic species (such as waders, herons, and gulls), as well as competition for nesting sites [13,14,15,16,17]. Additionally, research highlights soil alterations in areas with YLG colonies due to nutrient inputs, which influence vegetation composition and habitat structure [18,19,20,21,22].

The organic matter contribution from hundreds of *L. michahellis* individuals, which have been roosting on the dikes separating the salt ponds since the early 1990s, represents a significant nutrient increase in the soil (guano), producing changes in the chemical composition of the substrate and favoring alterations in the composition of vegetation [18,19,20]. This effect is more intense in areas of the saltworks where the pond separation dikes were not built or repaired using saline substrate material from the heating ponds.

Due to its population growth, ability to alter insect and local flora communities [21], and predation on vertebrate fauna, the YLG has been considered capable of modifying ecosystem dynamics and structure [23,24,25,26,27]. Regarding local fauna predation, it is worth noting that YLG reproduction in the Salinas de San Pedro del Pinatar, as in the Mediterranean region of the Iberian Peninsula, begins in March, with most clutches laid during the first half of April [28]. In contrast, other species of Laro-limicolae begin their breeding season a month later or, upon returning from their wintering grounds in Africa, find their territories occupied by YLG colonies, which they avoid due to the gulls’ predation on their chicks. This forces them to establish colonies in more marginal areas exposed to human presence and may even trigger extinction processes [21].

In some areas, management and nest control programs for YLG colonies have been implemented through partial elimination and/or eradication and have been considered successful by some authors [23,25,29,30]. In contrast, others consider these efforts as of limited efficacy or having a short-term scope, as they often focus on alleviating local consequences without addressing the underlying causes or understanding the demographic consequences of the actions [10,28,31,32].

Nevertheless, there is a lack of comprehensive studies analyzing the environmental, social, and economic consequences of the increase in the population of YLGs in a territory. Additionally, there are no published works evaluating the long-term results of management actions on the reproduction of YLGs, such as those carried out jointly in the Salinas de San Pedro del Pinatar. The objectives of this study were the following: (1) to identify the effects of YLGs on nesting waterfowls, workers, and salt production activities, (2) to quantify the results of the control methods implemented by the Administration between 2000 and 2021, as well as deterrent actions by Salinera Española to prevent damage to salt production, and, finally, (3) to develop proposals for controlling the YLG population at its source by adopting measures that hinder its access to anthropogenic food sources, with special attention to organic matter in municipal solid waste landfills, marine aquaculture farms, and fishery discards.

## 2. Materials and Methods

### 2.1. Study Area

**Figure 1:**
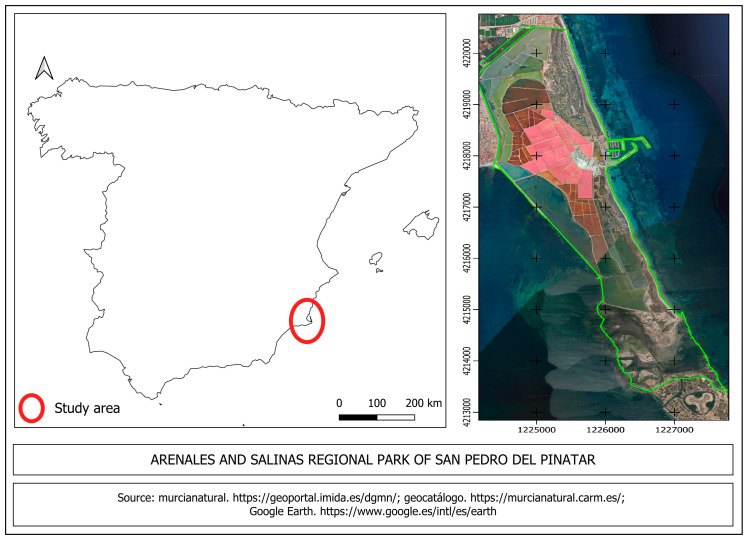
Boundaries of the Regional Park las Salinas y Arenales de San Pedro del Pinatar. The red circle indicates the landscape location. The pink colors observed in the image are due to different concentrations of salt. The green line represents the boundaries of the Regional Park. Accessed on 12 April 2024.

The Regional Park of the Salinas y Arenales de San Pedro del Pinatar encompasses an area of 856 hectares, situated in the northernmost coastal region of Murcia, Spain. This park is distinguished by its flat landscape and a diverse range of environments. Among these environments are salt ponds, which, depending on their function in the salt production process, are referred to as “storage, heating, or crystallization” pools (Figure 1).

Summers are hot, humid, and mostly clear; winters are long, cold, windy, and partly cloudy and remain dry throughout the year. The annual temperature ranges from 6 to 29 °C, rarely dropping below 2 °C or rising above 32 °C. The average annual precipitation is around 320 mm, with highly irregular annual and interannual distribution, including years with less than 200 mm and days when over 100 mm falls within a few hours. Nevertheless, it has high ambient humidity, with average values exceeding 70% saturation [33].

The natural park Salinas de San Pedro del Pinatar features a flat, sedimentary coastal morphology, characterized by dune formations and coastal sandy areas associated with wetlands but especially with extensive areas dedicated to salt pans and significant ecological systems of marshlands and other sedimentary ecosystems of the lagoon coastline: reedbeds, salt marshes, sandy areas, and beaches [34]. Over 200 species of vascular flora and 19 types of habitats of community interest have been described, of which 3 are considered a priority for conservation under Directive 92/43/EEC on the conservation of natural habitats and of wild fauna and flora (1150 Coastal Lagoons, 1510 Mediterranean Salt Steppes (Limonietalia), 2250 Coastal Dunes with *Juniperus* spp.) [35,36]. Among the fauna, birds stand out, with over 170 species recorded, 32 of which are listed in Annex I of the European Union Directive on the Conservation of Wild Birds (2009/147/EC), including 1–2% of the global breeding population of *Larus audouinii*, a species classified as vulnerable worldwide [2,37]. The Salinas de San Pedro del Pinatar is a well-established industrial operation with highly advanced technology, providing direct employment to around 50 workers. The salt produced is processed on-site, transformed into various types for different uses: food, industry, or de-icing, and packaged for distribution, primarily within the Iberian Peninsula [37].

The total surface area of all salt ponds adapted for salt production is 470 ha, with an annual production of approximately 80–100 thousand tons. The salt pans coexist with sandy areas and salt marshes typical of wetland areas and other sedimentary ecosystems of beaches and lagoon coastlines. Additionally, the park includes the Encañizadas, a pseudomareal area formed by shallow waters and channels between small islands connecting the Mar Menor with the Mediterranean Sea, harboring significant populations of waterfowls at the national, European, and even global levels [10].

Out of the 170 bird species with regular presence, 32 species stand out as they are listed in Annex I of the Birds Directive (2009/147/EC). The European Union designated the Salinas de San Pedro del Pinatar as a Special Protection Area for Birds (SPA); moreover, the park hosts significant populations in Spain of Audouin’s gull (*Ichthyaetus audouinii*), Sandwich tern (*Thalasseus sandvicensis*), slender-billed gull (*Larus genei*), Kentish plover (*Charadrius alexandrinus*), and common tern (*Sterna hirundo*) [38].

The sandy dikes separating the salt ponds are constructed and reinforced with sedimentary material from the basins, and their composition varies depending on whether they are “storage”, “heating”, or “crystallization” ponds. The bottom of the “heating” ponds has a substrate where materials with high NaCl, CaCO_3_, CaSO_4_, MgSO_4_, and MgCl_2_ content have accumulated. These compounds are entirely used to build and maintain the dikes separating the salt ponds, hindering vegetation growth and providing optimal habitats for the reproduction of various waterfowl colonies [10], habitats characterized by sandy or silty substrates, with little to no vegetation, and when present is typically halophytic [39].

### 2.2. Methodology Used

The results of nesting waterfowl and YLG population control censuses conducted in the Regional Park of the Salinas y Arenales de San Pedro del Pinatar (Murcia, Spain) between 1990 and 2021 have been analyzed. Additionally, winter bird censuses coordinated by the Association of Naturalists of the Southeast [5,40,41,42,43] have been considered.

The censuses of wintering waterbirds, including the YLG, are conducted in the Salinas de San Pedro del Pinatar, as well as worldwide, in mid-January, as part of the internationally coordinated censuses following the methodology proposed by Wetlands International [44]. In the Salinas de San Pedro del Pinatar, these censuses have been carried out continuously since 1990 along the paths that traverse the entire salt pan complex, early in the morning, using appropriate optical equipment, to count the total number of individuals present in the wetland [40]. On the other hand, censuses of breeding YLGs have been conducted since 1994, in mid-April, when the number of nesting pairs is highest. During these censuses, all sandy dikes separating the salt ponds are surveyed, and all nests with eggs are counted [5,44].

The breeding waterfowl censuses were conducted between April and July to cover the entire reproductive phenology of the species. For species that breed in the Salinas de San Pedro del Pinatar, it is necessary to survey the colonies on foot during the peak breeding period, when the first chicks begin to hatch, in order to count as accurately as possible the number of nests, eggs, and chicks [45].

For the analysis of the population trend of the YLG, the R statistical package “rtrim” [46] was used, which integrates the indices and trend models of the TRIM program (Trends and Indices for Monitoring data) [47]. This tool allows the calculation of population indices that represent the degree of interannual change. For trend analysis, the data must be organized so that the first available census of the species serves as the reference year for the model (value 1). The general trend is derived from “imputed” indices, meaning they are based on observed data. Thus, an index value of 2 would indicate that the population size of a species has doubled compared to the initial or reference year.

### 2.3. Measures to Control the Population of Yellow-Legged Gulls

These actions involve government interventions for biodiversity conservation, occupational safety, and salt production, combined with deterrent initiatives by the company managing the salt flats through the placement of deterrent elements to prevent damage in salt extraction and stacking areas, with the guidance and periodic evaluation of the measures adopted by expert ornithologists and the support of the Southeast Naturalists Association [48].

The salt workers periodically adapt the deterrent elements (life-sized ceramic owls in salt stacking areas and posts with nylon wire stretched to a height of 1 m around the perimeter of the crystallizer salt ponds) to specific needs as part of maintenance activities.

The Administration of the Region of Murcia initiated the control of YLG in the Salinas de San Pedro del Pinatar in the year 2000 with the aim of maintaining the main breeding areas for shorebirds free from YLG nests and preventing the risk of accidents with saltworks workers. During the breeding season, gulls engage in low flights, and there are even cases of attacks on individuals approaching their nests, as is the case in other YLG colonies in tourist areas (e.g., Benidorm Island) and urban zones, where low-altitude flights and incidents with people have been reported [49,50,51].

## 3. Results

### 3.1. Description and Analysis of the Area Affected

**Figure 2:**
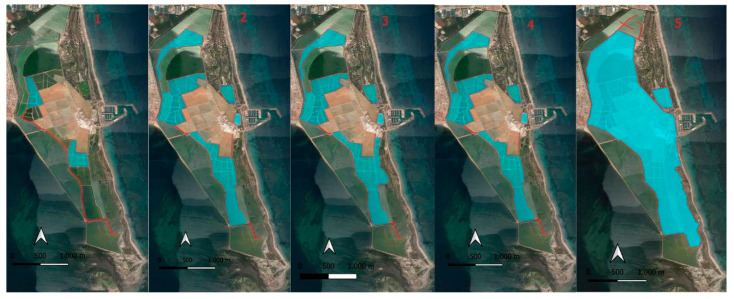
In cyan, area where nests were removed from *L. michahellis:* (1) 2000–2001, (2) 2002–2005, (3) 2006–2011, (4) 2012–2018, and (5) 2019–2021.

Population control of the breeding yellow-legged gull has been carried out since the year 2000 by eliminating nests and eggs between the last week of March and the first week of June along the dikes separating the salt ponds (Figure 2).

In the first year of yellow-legged gull control measures (year 2000), nests and eggs were removed along the most frequently used pathway by saltworks workers. This area also housed some colonies of common avocet (*R. avosetta*) and common tern (*S. hirundo*), which had experienced a reduction in nesting pairs in 1999 due to the increase in the yellow-legged gull population.

Over the years, the yellow-legged gull control system has been progressively improved; thus, in 2001, an attempt was made to puncture the eggs of nests located along the pathways, as these paths were traversed daily by saltworks employees. The results were not as expected, as many eggs still hatched. Therefore, this trial was not repeated [29]. Between 2002 and 2005, nest control was expanded to other frequently traveled areas within the Salinas, although breeding pairs were detected on dikes separating salt ponds that were not usually traversed by saltworks workers and were located at a certain distance from colonies of other waterfowls. From 2006 to 2018, the control area for the YLG was expanded again to include all accessible pathways within the Salinas, as well as some dikes separating salt ponds located next to the beaches south of the port. Finally, between 2019 and 2021, as part of the LIFE-SALINAS Project (LIFE17 NAT/ES/000184), comprehensive actions were taken on all pathways and dikes separating salt ponds within the Salinas de San Pedro del Pinatar. This included crystallization ponds, dikes separating salt ponds, and dunes parallel to the beaches south of the port.

### 3.2. Evolution of the Population of the YLG

**Figure 3:**
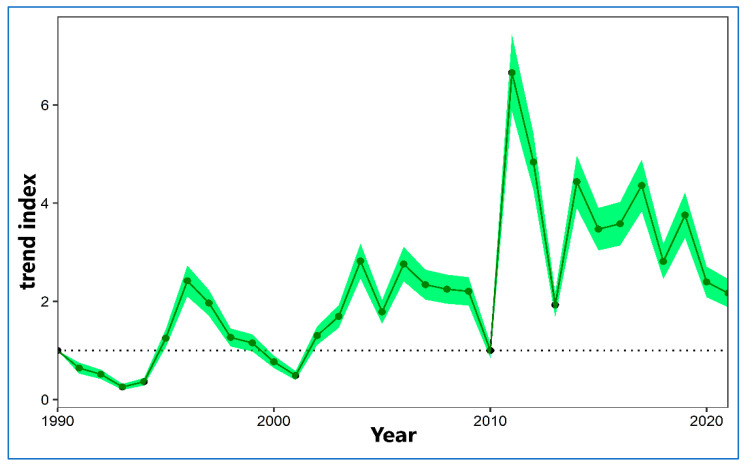
Trend of the wintering population of *L. michahellis* in the Regional Park of the Salinas y Arenales de San Pedro del Pinatar (1990–2021). The dotted line indicates the minimum growth index within the latest analyzed period (2010–2021).

**Figure 4:**
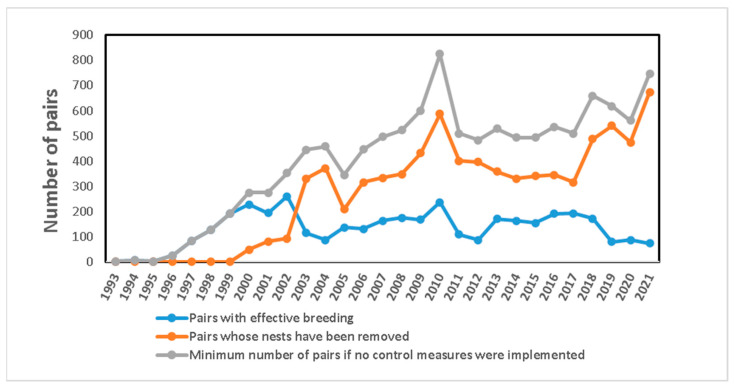
Number of pairs in the Reginal Park Las Salinas y Arenales de San Pedro del Pinatar (1993–2021).

The wintering population of the YLG in the Regional Park of the Salinas y Arenales de San Pedro del Pinatar shows a significant trend with a strong increase (Figure 3). The average population has been on the rise since the early 1990s when the wintering population was around 100–200 individuals, reaching a peak of 2113 individuals in 2011, from when the population was maintained or slightly decreased thus far (1990–1999: average 345 individuals; 2000–2009: average 578 individuals; 2020–2021: average 628 individuals) (Figure 4).

**Figure 5:**
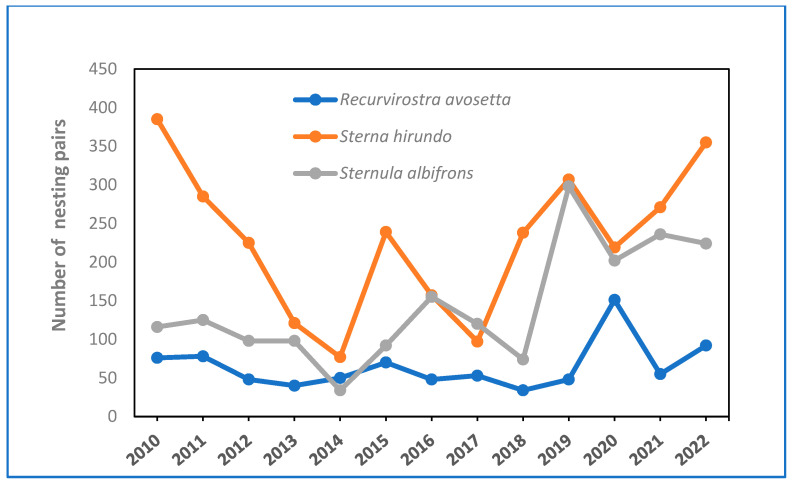
Evolution of the number of nesting pairs of *R. avosetta*, *S. hirundo*, and *S. albifrons* (2010–2022).

In the Salinas de San Pedro del Pinatar, two pairs of YLGs bred for the first time in 1993, showing a growing trend until reaching 227 pairs in the year 2000. From 2000 onwards, control measures for YLG were initiated, resulting in a reduction in the number of pairs with successful reproduction, ranging between 73 and 237 pairs. Meanwhile, the number of nests and their eggs eliminated varied, with a minimum of 48 nests removed in 2000 and a maximum of 588 nests removed in 2010 (Figure 5).

### 3.3. Landscape and Environmental Impacts

Although quantitative evaluations of habitat composition variations have not been conducted in the Salinas de San Pedro del Pinatar to date, the analysis of historical aerial photographs for the period 1990–2022 reveals a notable development in vegetation coverage and density. In some sectors where there was little to no vegetation decades ago, the coverage has increased to over 75% of the surface and, in some cases, even 100% of the total area. This represents a significant loss of optimal habitat for the reproduction of waterfowl, which relocate their breeding colonies to areas with less than 50% vegetation coverage.

Nesting waterfowl censuses conducted in the Regional Park of the Salinas y Arenales de San Pedro del Pinatar between 1994 and 2021 indicate that the eight nesting species listed in Annex I of Directive 2009/147/EC of 30 November 2009, concerning the conservation of wild birds (Birds Directive), namely *Himantopus himantopus*, *Recurvirostra avosetta*, *Charadrius alexandrinus*, *Ichthyaetus audouinii*, *Gelochelidon nilotica*, *Sterna hirundo*, *Sternula albifrons*, and *Thalasseus sandvicensis*, use the separation dikes of salt ponds with sparse vegetation coverage, as well as islets and beaches in the Encañizadas area, which are not frequented by people and/or domestic animals, as nesting sites.

Figure 5 shows the number of nesting pairs of three species from 2010 to 2022. A sharp decline was observed from 2010 to 2014 in the breeding colony of *S. hirundo*, dropping from nearly 400 pairs in 2010 to a minimum of 77 pairs in 2014 and 97 pairs in 2017. Similarly, *S. albifrons* fluctuated comparably, decreasing from 125 pairs in 2011 to 34 pairs in 2014 and 74 pairs in 2018. Likewise, *R. avosetta* declined from 78 pairs in 2011 to a minimum of 40 pairs in 2013, possibly due to the reduction in suitable nesting habitat as well as pressure from *L. michahellis*. Starting in 2019, *L. michahellis* control measures were implemented across all the salt flats, leading to an increasing trend in the number of pairs of the three species, with fluctuations during the 2019–2022 period ranging from 355 to 202 pairs for *S. hirundo*, 298 to 202 pairs for *S. albifrons*, and 151 to 49 pairs for *R. avosetta* (Figure 5).

**Figure 6:**
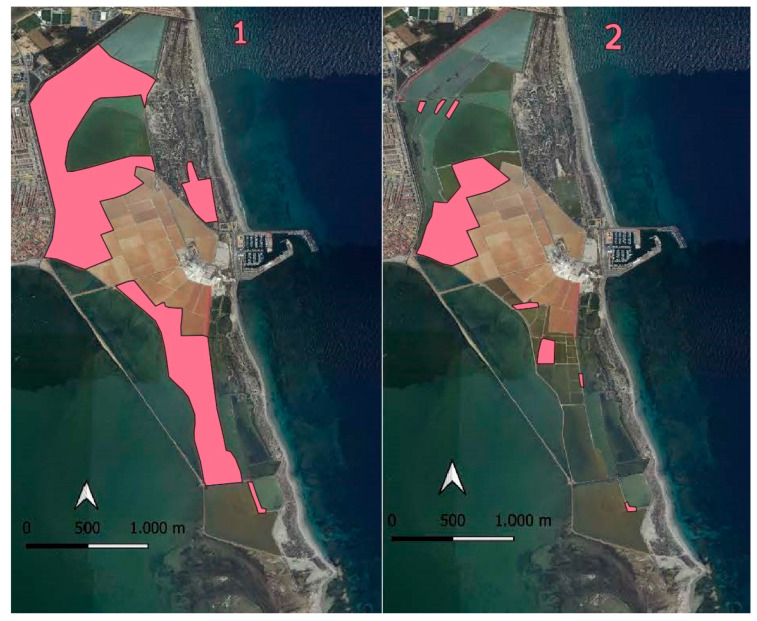
Area (in pink) occupied by colonies of breeding waterfowls: (1) in 1994 and (2) in 2015.

In 1994, a study was conducted to document the distribution of nesting waterfowls in the Regional Park of Salinas y Arenales de San Pedro del Pinatar and the Encañizadas area. At that time, waterfowls occupied a scattered area of 406 hectares, indicating a wide availability of suitable breeding habitat. However, when the study was repeated in 2015, a significant change in the distribution of these habitats was observed. Using maps to locate waterfowl colonies, it was found that the breeding habitat had become concentrated in just 85.4 hectares. This remarkable 79% reduction in suitable nesting habitat for waterfowls over 21 years reflects a significant deterioration in ecosystem conditions (Figure 6).

The Regional Park has experienced an increase in the breeding population of YLG since 1993 when 2 pairs bred, up to 191 pairs in 1999, with negative effects on several colonial waterfowl species. Notably, there was a 55.6% reduction in the nesting population of *S. hirundo* compared to the 1992–1995 average. Meanwhile, other species such as *R. avosetta*, *Ch. alexandrinus*, and *S. albifrons* relocated their nests to other areas within the protected space, sometimes in vulnerable zones accessible to humans and predators like rats, cats, and dogs.

Out of the 85.4 ha of suitable habitat for waterfowl reproduction in 2015, 71.6 ha (83.9%) are patches of warm puddles, 3.6 ha (4.2%) correspond to storage puddles, and 10.2 ha (11.9%) are in the intertidal zone of the Encañizadas. Therefore, the majority of waterfowl colonies have relocated their nests, forming large colonies near patches of warm puddles.

As vegetation covers the patches separating the “storage” puddles, the optimal surface for waterfowl reproduction decreases. This leaves the patches separating the “warm” puddles available for waterfowls, where salt management is more intense, and the substrate is composed of precipitated CaSO_4_, MgSO_4_, and MgCl_2_ salts that hinder vegetation growth chemically. Meanwhile, in the Encañizadas area, the available habitat for waterfowls is reduced to the zone with barely any land and/or sand deposits that allow vegetation growth, as well as the intertidal zone that emerges during the summer and remains submerged the rest of the year.

The breeding season of the YLG begins with nest construction in mid-March on the patches separating the saline puddles covered by halophytic vegetation, especially *Suaeda* spp. and *Arthrocnemum* spp. As the breeding period progresses and the number of YLG pairs increases, they install their nests in saline patches with a lower proportion or no vegetative cover, occupying territories that were used by other colonial waterfowls such as *R. avosetta*, *S. hirundo*, *S. albifrons*, etc., until the early 21st century. The reproductive phenology of these species begins in late April; so, upon reaching the breeding areas, they found them occupied by colonies of the YLG, a predatory species on their offspring. Consequently, they either abandoned the San Pedro del Pinatar Salinas or used areas far from YLGs, often in locations easily accessible to humans and predators.

Since 2 pairs first bred in the Salinas de San Pedro del Pinatar in 1993, YLG increased to 191 pairs by 1999. This growth has had negative impacts on various species of colonial waterfowls. In particular, a 55.6% decline was observed in the nesting population of *S. hirundo* compared to the 1992–1995 average. Additionally, other species such as *R. avosetta*, *Ch. alexandrinus*, and *S. albifrons* have relocated their nests to different areas within the protected space, sometimes in vulnerable zones accessible to predators such as rats, cats, and dogs.

In 2015, out of the 85.4 hectares of suitable breeding habitat for waterfowls, 83.9% (71.6 ha) consisted of warm ponds, 4.2% (3.6 ha) corresponded to storage ponds, and 11.9% (10.2 ha) was located in the intertidal zone of the Encañizadas. As a result, most waterfowl colonies have relocated their nests, forming large aggregations near these warm ponds.

As vegetation covers the areas between the storage ponds, the optimal surface for waterfowl reproduction is reduced. This leaves available the areas between the “warm” ponds, where salt management is more intense, and the substrate consists of salts such as CaSO_4_, MgSO_4_, and MgCl_2_, which negatively affect vegetation growth. In the Encañizadas area, the available habitat for waterfowls is limited to areas with sparse soil or sand accumulation that allows plant growth, as well as the intertidal zone, which emerges during the summer and remains submerged for the rest of the year.

### 3.4. Social and Economic Impacts

The damages are localized in the sanitary risk due to the introduction of various germs, such as *Listeria* and *Salmonella* sp., that seagulls obtain from the landfills where they feed and transfer to the salt pans, potentially affecting wildlife [17,18,20,36]. The entry occurs through two pathways: on one hand, hundreds of resident individual YLGs throughout the year perch to spend the night on the earth and sand dykes separating saline puddles, on the salt piles, and during the months from September to November, also on the crystallizer ponds once they have dried up for salt extraction, carrying both organic and inorganic residues obtained from their feeding areas, mainly landfills. All of this, combined with excrement, forced the salt-producing company to discard the first layer of salt, located on the surface of the crystallizer ponds. On the other hand, it was common to observe at dawn, apparently to obtain condensed water during the night, up to 120 YLGs perched and pecking at plastic salt bags stacked next to the industrial zone warehouses, waiting to be loaded onto trucks for distribution to shopping centers. The bags pecked by the seagulls had to be removed before the arrival of the trucks, resulting in annual losses estimated by Salinera Española at around EUR 120,000, implementing the preventive measures outlined in Section 3.5, while awaiting the adoption of necessary measures in urban solid waste landfills, aquaculture farms, and fishery discards, which would help reduce the global population of YLGs.

Regarding the safety of the workers of the salt-producing company, YLGs even placed their nests on the sides of transit roads, making low flights with intimidatory purposes over those who approached too close to their nests during the period when the eggs began to hatch and until the moment when the chicks could fly. In the years 1999 and 2000, the seagulls caused two accidents with salt workers due to motorcycle falls during their travels to carry out their routine tasks of regulating the salt concentration in the salt pans and various maintenance works.

### 3.5. Management of the Population of YLGs

The management of YLG nests is yielding the following results: (1) since 2002, there have been no incidents between saltworks workers and YLGs, (2) recovery of the population of *S. hirundo*, (3) return to the usual breeding areas of some waterfowl species of conservation interest (*Ch. alexandrinus*, *R. avosetta*, *S. hirundo*, and *S. albifrons*), and (4) facilitating the colonization of two waterfowl species that have few breeding locations in Europe: *I. audouinii* and *T. sandvicensis*.

The availability of space without YLG colonies could be one of the reasons that has facilitated the colonization of species listed in Annex I of the European Union Directive on the Conservation of Birds (Directive 49/147/EC), with relevant populations at the national, European, and even global level. Some of these species show a decreasing global trend, such as *S. sandvicensis*, which began breeding in 2008 in the Regional Park of Salinas y Arenales de San Pedro del Pinatar, while others have stable populations, such as *I. audouinii*, which also began breeding in the Salinas in 2010 (Table 1).

### 3.6. Actions to Minimize Damage in Salt Production

Salinera Española has placed life-sized ceramic owls in an open area where salt bags are stacked, located next to the buildings where the salt is packaged. This area experiences constant movement of people and vehicles during working hours, with the YLG being the only bird species present in the area. This technique, along with similar methods, has been tested in various studies with varying degrees of success [53,54,55]. Additionally, it is a harmless deterrent for YLGs and the least invasive option in the context of a protected natural area compared to other methods, such as pyrotechnics, distress calls, the use of falcons, or the application of lethal and non-lethal ammunition [13,56,57], which are typically used in areas with high concentrations of gulls, such as landfills, or in locations with significant safety risks for people, such as airports or fish farming facilities.

**Figure 7:**
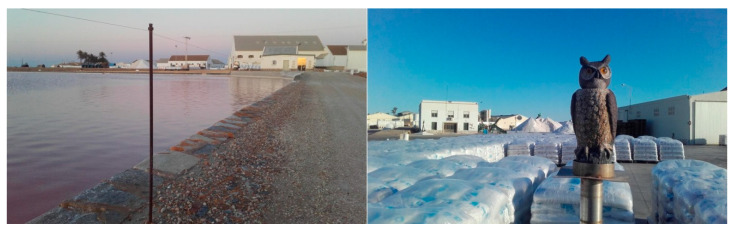
Nylon thread around a saltwater puddle and a life-sized ceramic owl.

On the other hand, posts with tensioned nylon threads, approximately 1 m high, have been installed around the entire perimeter of the crystallizer ponds. This prevents hundreds of seagulls from perching on the salt bags (particularly between late September and November), where they would otherwise leave food remnants from the landfills they use as feeding grounds, along with a layer of droppings (Figure 7). Due to the effectiveness of these deterrent measures, they have been adopted as routine tasks integrated into the standard activities of salt production.

## 4. Discussion

The article presents a comprehensive analysis of the impact of the YLG on the ecosystems of the Salinas de San Pedro del Pinatar, a phenomenon that coincides with observations made by other authors in different areas [18,19,20], as well as its effects on salt production and local biodiversity. Throughout the 20th century, the population of this species has experienced remarkable growth, which has resulted in a series of ecological and socioeconomic consequences, leading to the adoption of various control measures.

Regarding the ecological impact, the YLG has proven to be an effective predator, negatively affecting the populations of other gulls and waders that breed in the salt flats, as supported by different observations [21,31]. Competition for breeding territory and direct predation on nests and chicks have led to a decline in certain species of breeding gulls and waders.

Furthermore, the presence of the YLG has impacted salt production. The accumulation of guano and other organic waste, combined with the behavior of the gulls, has led to economic losses for the salt production company, which were calculated at EUR 120,000, emphasizing the need for strategies that not only protect biodiversity but also safeguard the economic interests of the salt industry.

Since 2000, various control measures have been implemented to manage the gull population, including the removal of nests and the installation of deterrent devices. However, the effectiveness of these measures must be continuously assessed, and it is crucial to make adjustments based on observation and analysis of results.

This article suggests that the reduction in anthropogenic food sources, such as landfills and other sources, could be a long-term solution to address this issue in a comprehensive manner.

## 5. Conclusions

The growth of the breeding population of the YLG in the Regional Park of Salinas and Arenales de San Pedro del Pinatar has put the diversity of breeding waterfowl species at risk due to nest predation, as well as habitat alteration caused by guano deposition. The control measures implemented since the year 2000 have proven effective in reducing the YLG breeding population and in the recovery of several displaced waterfowl species. The removal of nests and eggs has allowed species such as *S. hirundo* and *R. avosetta* to reclaim their traditional breeding areas, highlighting the importance of active management in the conservation of local biodiversity. However, the loss of suitable breeding habitat for waterbirds has reached 79% over the past 21 years, limiting nesting options. Additionally, the interaction between the YLG and salt industry workers continues to pose a risk to their physical safety, emphasizing the need to maintain management strategies that minimize these potential conflicts. The global solution to the problems caused by the YLG population in the Salinas de San Pedro del Pinatar lies in preventing or restricting access to anthropogenic food sources, particularly urban solid waste landfills, aquaculture farms, and fishery discards. It is proposed to maintain the control measures for the YLG, with future adjustments as needed, as long as the breeding population continues to pose a threat to the conservation of nesting larid and wader populations, as well as to the safety of workers and salt production.

## Figures and Tables

**Table 1:** Global, European, Spanish, and regional importance of nesting waterfowl in the Regional Park.

	Global Trend (IUCN, 2025) [2]	European Trend (UICN, 2025) [2]	Number of Pairs 2010–2020	Population (%)			
				Murcia Region	Spain	European Union	World
*Recurvirostra avosetta*	Unkown	Decreasing	55.9	99	1–2	-	-
*Charadrius alexandrinus*	Decreasing	Decreasing	59.8	61	1–2	-	-
*Ichthyaetus audouinii*	Steady	Decreasing	344	100	2–3	2–3	2–3
*Gelochelidon nilotica*	Decreasing	Steady	237	100	3–4	1–2	-
*Sterna hirundo*	Unknown	Unknown	282	99	11–12	-	-
*Sternula albifrons*	Decreasing	Decreasing	216	100	5–6	0.5	-
*Thalasseus sandvicensis*	Steady	Increasing	286	100	5–6	-	-

Source: Work from Molina [5], SEO/BirdLife [52], and IUCN [2].

## Data Availability

All data will be made available under reasonable request to the corresponding author.
